# Preoperative malnutrition predicts poor early immune recovery following gynecologic cancer surgery: a retrospective cohort study and risk nomogram development

**DOI:** 10.3389/fimmu.2025.1681762

**Published:** 2025-10-15

**Authors:** Xingyu Sun, Lan Li, Lijuan He, Shaohua Wang, Zhiling Pan, Dan Li

**Affiliations:** ^1^ Department of Gynecology, The Affiliated Traditional Chinese Medicine Hospital, Southwest Medical University, Luzhou, Sichuan, China; ^2^ Department of Pathology, The Affiliated Hospital, Southwest Medical University, Luzhou, China; ^3^ Department of Health Management Center, The Affiliated Hospital, Southwest Medical University, Luzhou, Sichuan, China; ^4^ Department of Operating Room, Affiliated Hospital of Youjiang Medical University for Nationalities, Baise, China; ^5^ Key Laboratory of Molecular Pathology in Tumors of GuangxiHigher Education Institutions, Affiliated Hospital of Youjiang Medical University for Nationalities, Baise, China; ^6^ Department of Oncology, The Affiliated Hospital, Southwest Medical University, Luzhou, China; ^7^ Sino-German Gynecologic Oncology Center, The Affiliated Hospital, Southwest Medical University, Luzhou, China

**Keywords:** malnutrition, immune recovery, gynecologic oncology, lymphocyte, nomogram

## Abstract

**Background:**

Malnutrition is prevalent in patients undergoing gynecologic cancer surgery and may compromise postoperative immune competence. However, its specific association with early immune recovery remains unclear, and validated predictive tools are lacking.

**Methods:**

This retrospective cohort study included 1,245 women who underwent curative surgery for cervical, endometrial, or ovarian cancer between March 2021 and September 2023. Preoperative nutritional status was assessed using the Patient-Generated Subjective Global Assessment (PG-SGA), and patients were stratified into well-nourished and malnourished groups. Poor immune recovery was defined as lymphocyte count <1.0 ×10^9^/L on postoperative day 3 (POD3). Multivariate logistic regression was used to identify independent predictors, and a nomogram was developed and internally validated using ROC analysis, calibration curve, and decision curve analysis (DCA).

**Results:**

Malnourished patients had a significantly higher risk of poor immune recovery (36.6% *vs*. 16.1%, P < 0.001) and postoperative complications. In multivariate analysis, malnutrition (adjusted OR: 2.41; 95% CI: 1.82–3.22), low BMI, anemia, elevated CRP, advanced FIGO stage, open surgery, preoperative lymphopenia, and older age were independently associated with poor immune recovery. The final model demonstrated good discrimination (AUC = 0.821; 95% CI: 0.798–0.845) and clinical utility. The nomogram provides individualized risk estimates to guide perioperative immunonutrition strategies.

**Conclusion:**

Malnutrition is an independent risk factor for impaired early immune recovery after gynecologic cancer surgery. Our predictive model offers a clinically applicable tool to identify high-risk patients and support personalized perioperative management. Future prospective validation is warranted.

## Introduction

Gynecologic malignancies—including cervical, endometrial, and ovarian cancers—remain significant contributors to global cancer morbidity and mortality among women (1, 2). Surgery is the cornerstone of curative treatment for these cancers; however, postoperative recovery is increasingly recognized as being influenced not only by tumor burden and surgical technique but also by the patient’s nutritional and immunologic status (3, 4).

Preoperative malnutrition, frequently underdiagnosed in gynecologic oncology patients, has been associated with increased postoperative complications, prolonged hospitalization, delayed bowel function, and reduced survival (5–7). Nutritional deficits impair systemic immune function and exacerbate the inflammatory response to surgical stress, thus compromising early postoperative recovery (8, 9). Tools like the Patient-Generated Subjective Global Assessment (PG−SGA) have shown utility in identifying malnourished patients (10, 11), but they are rarely integrated into risk stratification models for immune outcomes in this population.

Early immune recovery, often represented by lymphocyte rebound within the first 72 hours after surgery, plays a critical role in tissue healing, infection resistance, and downstream oncologic outcomes (12). Persistent postoperative lymphopenia has been associated with increased risk of infection, delayed wound healing, and impaired host-tumor immune surveillance (13). However, despite its clinical relevance, few studies have systematically evaluated predictors of early immune recovery following gynecologic cancer surgery, and no validated clinical models currently exist to estimate this risk.

Immunonutrition—targeting both macro- and micronutrients essential to immune function—has emerged as a promising strategy to support postoperative recovery in oncology (14). Trials in gastrointestinal and head-and-neck cancers have demonstrated improved lymphocyte responses and reduced complication rates with perioperative immunonutrition (15). Yet, evidence in gynecologic oncology remains limited and inconsistent (16). Importantly, most interventions have not been individualized based on baseline immune or nutritional risk, limiting their efficacy and implementation (17).

Given this gap, the development of a predictive model capable of identifying patients at high risk of poor immune recovery would offer critical clinical utility. Such a model could guide tailored immunonutritional interventions, inform ERAS protocols, and help optimize perioperative immune management strategies.

In this retrospective cohort study of 1,245 patients undergoing curative surgery for gynecologic malignancies, we aimed to investigate whether preoperative malnutrition—as assessed by the PG−SGA—predicts poor early immune recovery, defined by persistent lymphopenia on postoperative day 3. We further sought to develop and internally validate a risk prediction nomogram incorporating nutritional, inflammatory, oncologic, and surgical factors to enable individualized immune risk assessment and guide future clinical decision-making.

## Methods

### Study design and setting

This retrospective cohort study was conducted at a tertiary academic hospital in Southwest China. Consecutive patients undergoing primary surgery for gynecologic malignancies between March 2021 and September 2023 were identified through the hospital’s centralized electronic medical database, which is updated in real time and regularly audited for clinical quality control. The study protocol followed the Declaration of Helsinki and was approved by the Institutional Review Board (IRB). Given the retrospective design and anonymized data collection, the need for informed consent was waived.

### Patient selection

Eligible patients were women aged ≥18 years with histologically confirmed cervical, endometrial, or ovarian cancer who underwent either minimally invasive or open surgery with curative intent. Exclusion criteria included: (1) neoadjuvant chemotherapy or radiotherapy; (2) autoimmune diseases or chronic immunosuppressive therapy; (3) blood transfusion within 72 hours before POD3; (4) incomplete perioperative records; and (5) loss to follow-up before POD3. All data were screened and cross-validated by two independent researchers using a predefined data extraction protocol.

### Nutritional status assessment

Preoperative nutritional status was evaluated using the validated Patient-Generated Subjective Global Assessment (PG-SGA), administered by trained registered dietitians or certified oncology nurses within 7 days before surgery. Patients were categorized as well-nourished (PG-SGA A) or malnourished (PG-SGA B or C) according to established scoring criteria. This classification served as the primary stratification variable.

### Data collection and variable definitions

Demographic, clinical, and surgical data were extracted from electronic medical records. Laboratory markers included preoperative and postoperative day (POD) 1 and 3 measurements of serum albumin, hemoglobin (Hb), lymphocyte count, C-reactive protein (CRP), and interleukin-6 (IL-6, if available). IL-6 data were collected in a predefined subset of patients based on institutional immunologic monitoring protocols and were used for sensitivity analyses, not included in the main model.

The primary outcome was poor immune recovery, defined *a priori* as a total lymphocyte count <1.0 ×10^9^/L on POD3, based on published guidelines and internal expert consensus. This threshold reflects clinically meaningful perioperative immunosuppression, as persistent lymphopenia at 72 hours after surgery has been associated with higher risks of infectious complications, delayed wound healing, and impaired antitumor immune surveillance. Additional recovery indicators included: (1) ≥30% CRP reduction (POD1–POD3), (2) serum albumin increase ≥2 g/L (POD1–POD3), and (3) NLR <5.0 on POD3. Composite immune recovery was defined as fulfilling at least 3 of these 4 criteria, based on prior literature and expert consensus in perioperative immunology.

Postoperative complications were graded using the Clavien–Dindo classification and adjudicated independently by two attending gynecologic oncologists. Infectious complications included documented surgical site infections (SSI), pneumonia, or urinary tract infections (UTI). Recovery metrics—such as time to ambulation ≥6 hours/day, bowel function return, and length of stay—were recorded per institutional enhanced recovery after surgery (ERAS) protocol.

### Statistical analysis

Continuous variables were reported as mean ± standard deviation (SD) or median with interquartile range (IQR) and compared using Student’s t-test or Mann–Whitney U test, as appropriate. Categorical variables were analyzed using chi-square or Fisher’s exact test. Multivariate logistic regression was used to identify independent predictors of poor immune recovery, employing backward stepwise selection with consideration of both clinical relevance and statistical significance (threshold for entry: P < 0.10). All variables with univariate P < 0.10 and clinical relevance were considered for model inclusion.

Model discrimination was evaluated using the area under the receiver operating characteristic curve (AUC), and calibration was assessed via the Hosmer–Lemeshow goodness-of-fit test. To mitigate multicollinearity, variance inflation factors (VIFs) were calculated, all <2. Internal validation was performed using 1,000-bootstrap resampling to ensure robustness and reduce overfitting.

A clinical nomogram was constructed from the final multivariate model. Predictive performance was evaluated by ROC curves, calibration plots, and decision curve analysis (DCA), with net benefit quantified across multiple risk thresholds.

### Data quality and bias control

All variables were cross-verified by two independent researchers blinded to outcomes. Any discrepancies were resolved by a third investigator. Complete case analysis was adopted, as the proportion of missing data was <5% across all variables. The study conforms to the STROBE (Strengthening the Reporting of Observational Studies in Epidemiology) guidelines.

## Results

### Baseline characteristics

**Table 1** presents the baseline characteristics of 1,245 patients stratified by preoperative nutritional status. Compared to the well-nourished group, malnourished patients were significantly older (58.1 ± 12.1 *vs*. 54.2 ± 11.4 years, P < 0.001) and had lower BMI (22.3 ± 4.1 *vs*. 24.5 ± 3.6 kg/m², P < 0.001). The malnourished group had a higher proportion of postmenopausal women, advanced-stage cancers (FIGO III–IV: 70.2% *vs*. 34.7%, P < 0.001), and ovarian cancer cases. They also had higher rates of comorbidities including anemia (46.3% *vs*. 23.6%, P < 0.001), hypertension, and diabetes. Laboratory indicators showed significantly lower serum albumin and lymphocyte counts and higher CRP levels in malnourished patients, suggesting a poorer systemic and immunologic baseline.

**Table 1:** Baseline characteristics of patients stratified by preoperative nutritional status (N = 1245).

Characteristic	Well-nourished (n = 712)	Malnourished (n = 533)	*P*-value
Age, years, mean ± SD	54.2 ± 11.4	58.1 ± 12.1	<0.001
BMI, kg/m², mean ± SD	24.5 ± 3.6	22.3 ± 4.1	<0.001
Menopausal status, n (%)			
– Premenopausal	265 (37.2%)	137 (25.7%)	<0.001
– Postmenopausal	447 (62.8%)	396 (74.3%)	
Type of cancer, n (%)	0.008		
– Cervical cancer	292 (41.0%)	204 (38.3%)	
– Endometrial cancer	224 (31.5%)	164 (30.8%)	
– Ovarian cancer	196 (27.5%)	165 (30.9%)	
FIGO stage, n (%)	<0.001		
– I–II	465 (65.3%)	159 (29.8%)	
– III–IV	247 (34.7%)	374 (70.2%)	
Comorbidities, n (%)			
– Hypertension	153 (21.5%)	145 (27.2%)	0.020
– Diabetes mellitus	96 (13.5%)	96 (18.0%)	0.038
– Anemia (Hb <110 g/L)	168 (23.6%)	247 (46.3%)	<0.001
Serum albumin, g/L, mean ± SD	39.4 ± 3.6	33.8 ± 3.9	<0.001
Lymphocyte count, ×10^9^/L, mean ± SD	1.68 ± 0.58	1.30 ± 0.59	<0.001
CRP, mg/L, median (IQR)	4.2 (1.8–9.4)	8.6 (3.2–17.3)	<0.001

BMI, body mass index; SD, standard deviation; FIGO, International Federation of Gynecology and Obstetrics; Hb, hemoglobin; CRP, C-reactive protein; IQR, interquartile range; POD, postoperative day; NLR, neutrophil-to-lymphocyte ratio; IL-6, interleukin-6; ICU, intensive care unit. Nutritional status was categorized based on the Patient-Generated Subjective Global Assessment (PG-SGA): well-nourished (PG-SGA A), malnourished (PG-SGA B or C). P-values were calculated using Student’s t-test, Mann–Whitney U test, or chi-square test, as appropriate.

### Perioperative immune and inflammatory marker dynamics

As shown in **Table 2**, both groups exhibited postoperative declines in lymphocyte count and albumin, and elevations in CRP and IL-6, consistent with expected inflammatory responses. However, malnourished patients had significantly lower lymphocyte counts and albumin levels, and higher NLR, CRP, and IL-6 concentrations at all time points (pre-op, POD1, and POD3; all P < 0.001). These findings indicate a more pronounced and prolonged inflammatory state and impaired immune recovery in malnourished individuals.

**Table 2:** Perioperative immune and inflammatory markers stratified by preoperative nutritional status.

Marker	Timepoint	Well-nourished (n = 712)	Malnourished (n = 533)	*P*-value
Lymphocyte count (×10^9^/L), mean ± SD	Pre-op	1.68 ± 0.58	1.30 ± 0.59	<0.001
POD1	1.05 ± 0.42	0.86 ± 0.41	<0.001	
POD3	1.26 ± 0.51	1.04 ± 0.48	<0.001	
NLR, median (IQR)	Pre-op	2.61 (1.82–3.69)	3.24 (2.15–4.52)	<0.001
POD1	8.12 (5.45–11.06)	10.67 (7.62–14.58)	<0.001	
POD3	4.73 (3.14–6.85)	5.91 (3.89–8.47)	<0.001	
Albumin (g/L), mean ± SD	Pre-op	39.4 ± 3.6	33.8 ± 3.9	<0.001
POD1	33.2 ± 3.8	29.4 ± 3.9	<0.001	
POD3	35.1 ± 3.7	31.2 ± 3.8	<0.001	
CRP (mg/L), median (IQR)	Pre-op	4.2 (1.8–9.4)	8.6 (3.2–17.3)	<0.001
POD1	68.5 (49.4–95.3)	80.7 (56.1–109.8)	<0.001	
POD3	37.4 (23.5–58.8)	48.5 (31.7–72.6)	<0.001	
IL-6 (pg/mL), median (IQR)*	Pre-op	7.3 (2.8–13.4)	10.5 (4.7–18.1)	<0.001
POD1	78.2 (55.6–113.4)	98.9 (67.4–136.1)	<0.001	
POD3	41.2 (26.8–64.3)	51.7 (30.9–74.1)	<0.001	

POD, postoperative day; SD, standard deviation; IQR, interquartile range; NLR, neutrophil-to-lymphocyte ratio; CRP, C-reactive protein; IL-6, interleukin-6; BMI, body mass index; FIGO, International Federation of Gynecology and Obstetrics; Hb, hemoglobin; ICU, intensive care unit.Nutritional status was defined by PG-SGA: well-nourished = PG-SGA A; malnourished = PG-SGA B or C. P-values represent between-group comparisons at each timepoint using independent t-test or Mann–Whitney U test. *IL-6 values were available in a subset of 412 patients according to institutional immunological monitoring protocols.

### Immune recovery on postoperative day 3

**Table 3** summarizes immune recovery outcomes on POD3. Lymphocyte recovery (≥1.0 ×10^9^/L) was achieved in 83.9% of well-nourished patients versus only 63.4% of malnourished patients (P < 0.001). Similar trends were observed for CRP resolution, albumin rebound, and normalized NLR. Among the subset with IL-6 data, a ≥40% decline from POD1 to POD3 was more frequent in well-nourished patients (77.2% *vs*. 62.6%, P = 0.002). The median number of immune recovery indicators met was higher in the well-nourished group (3 *vs*. 2), and composite immune recovery (≥3 indicators) occurred in 63.8% of well-nourished patients versus 38.7% of malnourished patients (P < 0.001), suggesting a clear association between nutritional status and immune competence.

**Table 3:** Immune recovery on postoperative day 3 according to preoperative nutritional status.

Immune Recovery Indicator	Well-nourished (n = 712)	Malnourished (n = 533)	*P*-value
Lymphocyte recovery (≥1.0 ×10^9^/L on POD3), n (%)	598 (83.9%)	338 (63.4%)	<0.001
CRP resolution (>30% decrease from POD1 to POD3), n (%)	516 (72.5%)	294 (55.2%)	<0.001
Albumin rebound (POD3 - POD1 ≥ +2 g/L), n (%)	438 (61.5%)	211 (39.6%)	<0.001
NLR <5.0 on POD3, n (%)	456 (64.0%)	267 (50.1%)	<0.001
IL-6 decrease >40% from POD1 to POD3*, n (%)	217/281 (77.2%)	82/131 (62.6%)	0.002
Number of recovery indicators met, median (IQR) (out of 4)**	3 (2–4)	2 (1–3)	<0.001
Composite recovery (≥3 indicators met), n (%)	454 (63.8%)	206 (38.7%)	<0.001

POD, postoperative day; CRP, C-reactive protein; IL-6, interleukin-6; NLR, neutrophil-to-lymphocyte ratio; BMI, body mass index; SD, standard deviation; FIGO, International Federation of Gynecology and Obstetrics; Hb, hemoglobin; ICU, intensive care unit; IQR, interquartile range. “Immune recovery” was assessed on POD3 based on the following four core indicators: (1) lymphocyte count ≥1.0 ×10^9^/L, (2) CRP decrease >30% from POD1, (3) serum albumin increase ≥2 g/L from POD1, and (4) NLR <5.0. IL-6 data were available in a subset of 412 patients (well-nourished: 281; malnourished: 131). Composite immune recovery was defined as meeting at least 3 of the 4 primary criteria. P-values were calculated using chi-square test or Mann–Whitney U test, as appropriate. Thresholds were defined based on literature and clinical consensus.

*Indicates that IL-6 measurements were available only in a predefined subset of 412 patients according to institutional immunological monitoring protocols.

### Postoperative clinical outcomes

**Table 4** details postoperative outcomes. Malnourished patients experienced significantly higher rates of overall complications (28.3% *vs*. 13.8%, P < 0.001), with an excess risk observed in both minor and major events. Infectious complications and delayed gastrointestinal recovery were also more common. Additionally, malnourished patients experienced longer hospital stays (13.1 ± 4.3 *vs*. 10.8 ± 3.7 days, P < 0.001), greater hospitalization costs, delayed ambulation, and higher 30-day readmission rates. These findings underscore the clinical consequences of poor nutritional status beyond laboratory immune markers.

**Table 4:** Postoperative clinical outcomes stratified by preoperative nutritional status.

Outcome	Well-nourished (n = 712)	Malnourished (n = 533)	*P*-value
Any postoperative complication, n (%)	98 (13.8%)	151 (28.3%)	<0.001
Minor (Clavien-Dindo Grade I–II)	69 (9.7%)	112 (21.0%)	<0.001
Major (Grade ≥ III)	29 (4.1%)	39 (7.3%)	0.018
Infectious complication (SSI, pneumonia, UTI), n (%)	51 (7.2%)	92 (17.3%)	<0.001
Delayed GI recovery (no flatus ≥72 h), n (%)	38 (5.3%)	66 (12.4%)	<0.001
ICU admission postoperatively, n (%)	8 (1.1%)	21 (3.9%)	0.006
Time to ambulation ≥6h/day, days, median (IQR)	2 (2–3)	3 (2–4)	<0.001
Time to bowel function recovery, days, mean ± SD	2.7 ± 1.1	3.5 ± 1.3	<0.001
Length of hospital stay, days, mean ± SD	10.8 ± 3.7	13.1 ± 4.3	<0.001
Total hospital cost, USD, median (IQR)	6,780 (6,020–7,810)	8,090 (7,130–9,270)	<0.001
30-day unplanned readmission, n (%)	19 (2.7%)	36 (6.8%)	0.001

ICU, intensive care unit; IQR, interquartile range; SD, standard deviation; GI, gastrointestinal; SSI, surgical site infection; UTI, urinary tract infection; POD, postoperative day; NLR, neutrophil-to-lymphocyte ratio; CRP, C-reactive protein; IL-6, interleukin-6; BMI, body mass index; FIGO, International Federation of Gynecology and Obstetrics; Hb, hemoglobin. Postoperative complica tions were categorized according to the Clavien-Dindo classification. Total hospital cost was recorded in Chinese Yuan and converted to USD using the average exchange rate during the study period (1 USD ≈ 6.5 RMB). Delayed GI recovery was defined as absence of flatus or bowel movement for ≥72 hours after surgery. Ambulation and GI recovery were documented according to institutional ERAS protocol. P-values were calculated using chi-square, t-test, or Mann–Whitney U test as appropriate.

### Independent predictors of poor immune recovery

**Table 5** displays the results of multivariate logistic regression identifying independent predictors of poor immune recovery (lymphocyte count <1.0 ×10^9^/L on POD3). Malnutrition was a strong predictor (adjusted OR: 2.41; 95% CI: 1.82–3.22; P < 0.001), along with BMI <18.5 kg/m², anemia, elevated preoperative CRP, advanced FIGO stage, ovarian cancer, open surgery, preoperative lymphopenia, and age ≥60 years (all P < 0.05). The model demonstrated good discrimination (AUC = 0.821; 95% CI: 0.798–0.845) and acceptable calibration (Hosmer–Lemeshow P = 0.26). These results confirm that nutritional and inflammatory status, alongside tumor burden and surgical factors, independently affect postoperative immune restoration.

**Table 5:** Multivariate logistic regression for predictors of poor immune recovery on postoperative day 3 (lymphocyte count <1.0 ×10^9^/L).

Variable Category	Variable	Adjusted OR (95% CI)	*P*-value
Nutritional status	Malnourished (*vs* well-nourished)	2.41 (1.82–3.22)	<0.001
	BMI <18.5 kg/m²	1.72 (1.18–2.51)	0.004
Hematologic/inflammatory	Anemia (Hb <110 g/L)	1.48 (1.10–2.00)	0.009
	Elevated pre-op CRP (>10 mg/L)	1.64 (1.22–2.19)	0.001
Tumor-related factors	FIGO stage III–IV (*vs* I–II)	1.59 (1.19–2.11)	0.002
	Ovarian cancer (*vs* cervical)	1.37 (1.00–1.88)	0.047
Surgical approach	Open surgery (*vs* minimally invasive)	1.46 (1.10–1.94)	0.009
Baseline immune status	Pre-op lymphocyte <1.0 ×10^9^/L	2.73 (2.01–3.70)	<0.001
Demographics	Age ≥60 years	1.31 (1.00–1.73)	0.049

Model performance: AUC = 0.821 (95% CI: 0.798–0.845); Hosmer–Lemeshow P = 0.26

OR, odds ratio; CI, confidence interval; BMI, body mass index; Hb, hemoglobin; CRP, C-reactive protein; FIGO, International Federation of Gynecology and Obstetrics; POD, postoperative day; NLR, neutrophil-to-lymphocyte ratio; IL-6, interleukin-6; ICU, intensive care unit; SD, standard deviation; IQR, interquartile range. Poor immune recovery was defined as lymphocyte count <1.0 ×10^9^/L on postoperative day 3 (POD3). All variables were selected based on backward stepwise logistic regression. Multicollinearity was assessed (VIF <2). Model discrimination was assessed using ROC (AUC), calibration by Hosmer–Lemeshow test, and clinical utility via decision curve analysis.

### Performance and visualization of the predictive model

**Figure 1:**
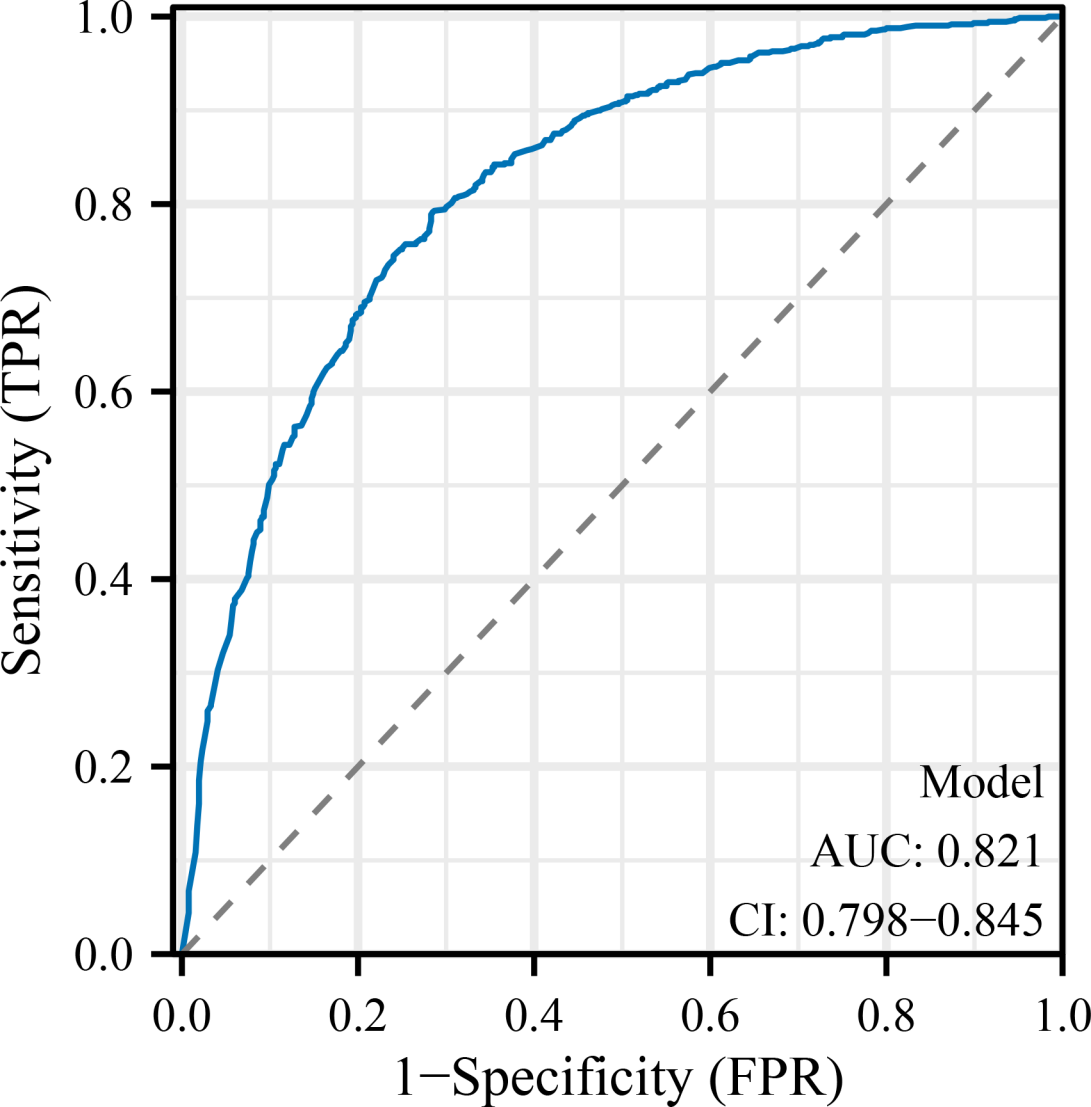
Receiver operating characteristic (ROC) curve for the predictive model. The ROC curve demonstrates the discrimination ability of the final logistic regression model in predicting poor immune recovery on postoperative day 3. The area under the curve (AUC) was 0.821 (95% CI: 0.798–0.845), indicating good model performance. POD, postoperative day; NLR, neutrophil-to-lymphocyte ratio; CRP, C-reactive protein; IL-6, interleukin-6; FIGO, International Federation of Gynecology and Obstetrics; ICU, intensive care unit; BMI, body mass index; SD, standard deviation; IQR, interquartile range; OR, odds ratio; CI, confidence interval.

**Figure 2:**
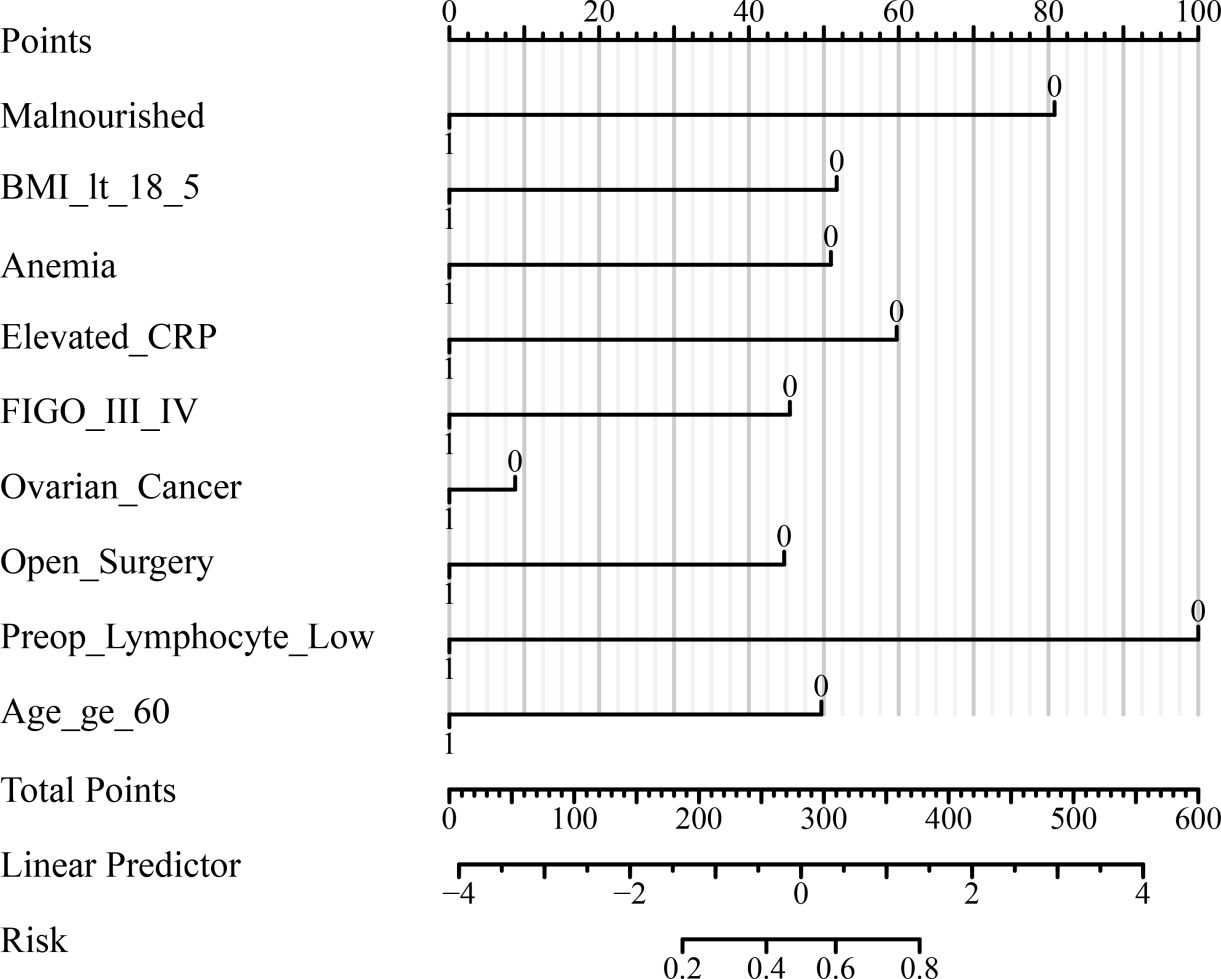
Nomogram for predicting poor immune recovery on postoperative day 3. The nomogram was developed based on the multivariate logistic regression model to estimate the individual risk of poor immune recovery (lymphocyte count <1.0 ×10^9^/L on POD3). Each predictor is assigned a corresponding score on the “Points” scale, and total points correspond to predicted probability at the bottom of the scale. POD, postoperative day; NLR, neutrophil-to-lymphocyte ratio; CRP, C-reactive protein; IL-6, interleukin-6; FIGO, International Federation of Gynecology and Obstetrics; ICU, intensive care unit; BMI, body mass index; SD, standard deviation; IQR, interquartile range; OR, odds ratio; CI, confidence interval.

**Figure 3:**
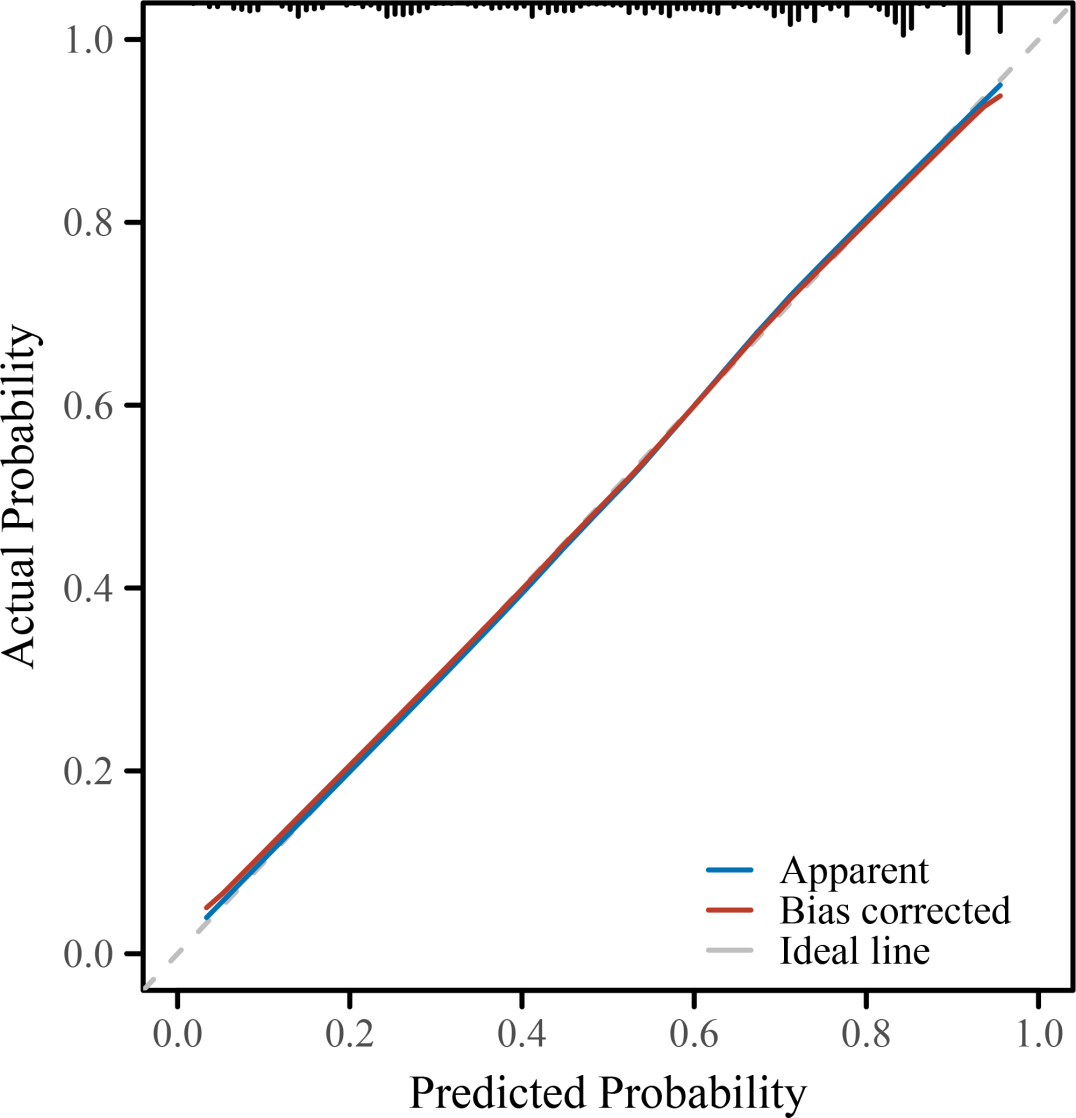
Calibration plot of the predictive model. The calibration curve compares the predicted probabilities with the observed outcomes of poor immune recovery on POD3. The bias-corrected line (via 1000-bootstrap resampling) closely aligns with the ideal line, indicating good agreement between predicted and actual risk. POD, postoperative day; NLR, neutrophil-to-lymphocyte ratio; CRP, C-reactive protein; IL-6, interleukin-6; FIGO, International Federation of Gynecology and Obstetrics; ICU, intensive care unit; BMI, body mass index; SD, standard deviation; IQR, interquartile range; OR, odds ratio; CI, confidence interval.

**Figure 4:**
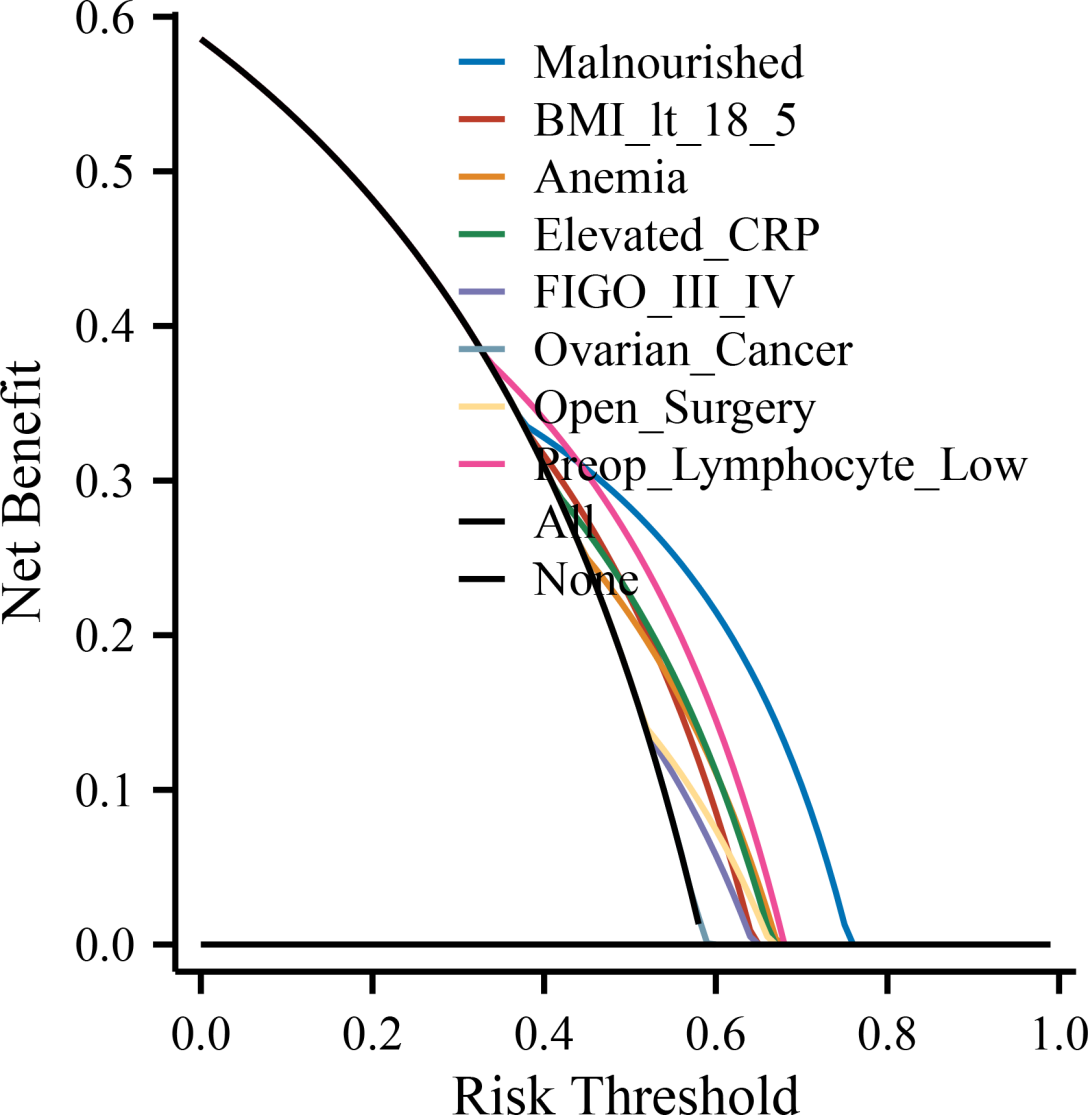
Decision curve analysis (DCA) for the predictive model. The DCA evaluates the clinical utility of the prediction model by estimating the net benefit across a range of threshold probabilities. The model shows greater net benefit than the “treat all” and “treat none” strategies within the clinically relevant range. POD, postoperative day; NLR, neutrophil-to-lymphocyte ratio; CRP, C-reactive protein; IL-6, interleukin-6; FIGO, International Federation of Gynecology and Obstetrics; ICU, intensive care unit; BMI, body mass index; SD, standard deviation; IQR, interquartile range; OR, odds ratio; CI, confidence interval.

To further evaluate and visualize the predictive performance of the multivariate logistic regression model, we conducted ROC analysis, nomogram construction, calibration assessment, and decision curve analysis. As shown in **Figure 1**, the receiver operating characteristic (ROC) curve demonstrated good discrimination of the final model, with an area under the curve (AUC) of 0.821 (95% CI: 0.798–0.845), indicating strong predictive ability for poor immune recovery on postoperative day 3. A nomogram incorporating all significant predictors from the final logistic regression (malnutrition, BMI <18.5, anemia, elevated CRP, advanced FIGO stage, ovarian cancer, open surgery, preoperative lymphopenia, and age ≥60) was developed to allow individualized risk estimation (**Figure 2**). Each predictor was assigned a score on a point scale, and the total score corresponds to the estimated probability of poor immune recovery. Model calibration was assessed using a bootstrap-corrected calibration curve (**Figure 3**), which showed close agreement between predicted and observed probabilities across the range of risk estimates, indicating good model calibration without significant overfitting. Finally, decision curve analysis (**Figure 4**) demonstrated that the predictive model provided greater net benefit across a range of clinically relevant risk thresholds compared to either “treat all” or “treat none” strategies. This suggests favorable clinical utility of the model in supporting perioperative immunological decision-making.

## Discussion

This study highlights the critical prognostic role of preoperative nutritional status in shaping early immune recovery among women undergoing curative gynecologic cancer surgery. Leveraging a large, well-characterized cohort and validated nutritional assessment via PG-SGA, we found that malnourished patients exhibited a significantly higher incidence of persistent lymphopenia on postoperative day 3 (POD3), delayed inflammatory resolution, and increased complication rates. The predictive nomogram constructed from these findings—integrating nutritional, inflammatory, oncologic, and surgical parameters—demonstrated strong discriminatory power (AUC = 0.821), suggesting its potential as a clinical decision-support tool in perioperative care.

Our findings substantiate and extend existing evidence linking malnutrition to postoperative vulnerability in cancer patients. While hypoalbuminemia and low BMI have previously been associated with increased morbidity and mortality in gynecologic oncology (18, 19), most prior research has not examined immune recovery as a distinct biological endpoint. By focusing on lymphocyte count on postoperative day 3 (POD3)—a clinically validated surrogate marker of immune function in the immediate postoperative period—our study contributes to the growing clinical understanding of host immune restoration following surgical stress. This approach is supported by recent evidence in spine surgery patients, where lower POD3 lymphocyte levels were significantly associated with increased risk of postoperative infections, reinforcing its prognostic utility in immunologic surveillance (20). Similarly, in minimally invasive thoracic surgery for non–small cell lung cancer, POD3 leukocyte and lymphocyte dynamics were shown to correlate with surgical stress intensity and early recovery outcomes, further validating this metric as a reliable indicator of immune recovery (21). Lymphocyte count <1.0 ×10^9^/L has been widely adopted in immune-oncology studies as a threshold indicating immunosuppression, particularly in the perioperative setting (22, 23). Our inclusion of complementary markers such as CRP, albumin kinetics, and NLR further strengthens the multidimensional assessment of postoperative immunologic recovery.

Importantly, our results revealed heterogeneity across tumor types. Malnourished patients with ovarian cancer—who represented a higher proportion of FIGO stage III–IV cases—demonstrated the poorest immune recovery rates. This aligns with previous studies demonstrating that preoperative malnutrition—often driven by extensive tumor burden and aggressive cytoreductive surgery—can compromise surgical outcomes through amplified nutritional stress and systemic inflammation. In particular, a recent multicenter cohort of ovarian cancer patients found that malnourished individuals were significantly more likely to undergo incomplete cytoreduction and experience worsened postoperative recovery (24). Thus, the prognostic effect of malnutrition may be particularly pronounced in high-stage ovarian cancer, warranting tailored preoperative intervention strategies in this subgroup. At the molecular level, metabolic reprogramming has also been implicated in treatment resistance, with adrenomedullin shown to induce cisplatin chemoresistance in ovarian cancer through glucose metabolism remodeling (25).

The biological plausibility of these findings is underpinned by a growing body of literature on the malnutrition-inflammation-immuno suppression axis. Protein-energy deficiency impairs lymphopoiesis, reduces thymic output, and disrupts antigen presentation through dendritic cell dysfunction (26). Simultaneously, micronutrient deficiencies—particularly zinc, selenium, and vitamin D—compromise T-cell proliferation, NK cell cytotoxicity, and cytokine signaling (27, 28). These effects are exacerbated in the perioperative setting by the acute-phase response, driven by IL-6 and other proinflammatory cytokines, which divert amino acids toward hepatic protein synthesis at the expense of peripheral immune function (29). This multifaceted immunologic compromise offers a compelling rationale for nutritional intervention strategies targeting both macronutrient repletion and immune modulation.

Immunonutrition, particularly formulations enriched with arginine, omega-3 fatty acids, and nucleotides, has shown promise in enhancing lymphocyte counts and reducing infections in gastrointestinal and head-and-neck cancer surgeries (30). Evidence in gynecologic oncology remains limited but encouraging. Ferrero et al. reported that perioperative immunonutrition improved CD8+ T-cell recovery and reduced infectious complications in patients undergoing surgery for advanced ovarian cancer (31). Our nomogram could serve as a risk-stratification tool to identify candidates most likely to benefit from such interventions, thereby improving cost-effectiveness and clinical outcomes.

From a global perspective, few predictive models exist to estimate early immune recovery in gynecologic surgery. Existing tools such as the Surgical Apgar Score or the ACS-NSQIP risk calculator offer general complication risk estimates but lack specificity for immune function or nutritional risk (32, 33). Recent oncology studies have similarly demonstrated the prognostic utility of nomogram-based approaches in colorectal, hepatobiliary, and pancreatic cancers, further supporting the methodological robustness and translational potential of our model (34–36). Compared to these, our model incorporates objective immunologic endpoints, uses widely available clinical data, and demonstrated high internal validity. Similarly, molecular signature-based prognostic models, such as ferroptosis-related signatures in gastric cancer, further highlight the translational relevance of integrating biologic markers into risk stratification (37).

These features enhance its potential for clinical translation, particularly in middle-income countries where nutritional risk is high and resource allocation must be strategic.

Despite its strengths, this study has several limitations. First, the retrospective design may introduce selection and information biases, although extensive cross-validation and standardized PG-SGA administration mitigate these concerns. Moreover, the large sample size and uniform preoperative nutritional assessment using a validated PG-SGA instrument help to reduce heterogeneity and strengthen the robustness of our findings. Second, the single-center setting may limit generalizability, necessitating external validation across diverse populations and health systems. Comparable large-scale studies in gastrointestinal oncology have shown that comorbid cardiovascular and metabolic diseases significantly influence long-term surgical outcomes, underscoring the need for broader validation of our findings (38). Third, IL-6 measurements were limited to a predefined subset based on institutional protocols. While informative for secondary analysis, our primary model used universally available biomarkers (CRP and lymphocyte count), enhancing clinical applicability. Fourth, the current model does not include long-term oncologic outcomes such as recurrence or survival. Whether early immune recovery mediates these endpoints remains an important area for future investigation.

Prospective validation of our nomogram is warranted, ideally through multicenter cohort studies encompassing different ethnic and geographic populations. Furthermore, randomized controlled trials should explore whether risk-stratified nutritional interventions based on the model can improve immune recovery and clinical endpoints. Finally, integration of more granular immune metrics—such as CD4/CD8 ratio, Treg counts, or immune gene expression profiles—could refine the model’s biological precision and clinical relevance.

In conclusion, preoperative malnutrition is a strong, independent predictor of impaired early immune recovery following gynecologic cancer surgery. Our internally validated prediction model offers a clinically accessible tool to identify high-risk individuals and tailor perioperative management. By bridging nutritional and immunologic assessment, this work provides a framework for future interventional studies and highlights the need for integrated nutrition-immunity strategies in oncologic surgery. In addition, the nomogram can be readily applied in routine clinical practice at the bedside using standard perioperative variables, enabling individualized risk estimation and targeted immunonutritional interventions.

## Data Availability

The raw data supporting the conclusions of this article will be made available by the authors, without undue reservation.
